# Antidiabetic activity of avocado seeds (*Persea americana* Mill.) in diabetic rats via activation of PI3K/AKT signaling pathway

**DOI:** 10.1038/s41598-022-07015-8

**Published:** 2022-02-21

**Authors:** Oluwafemi Adeleke Ojo, Jennifer Chidubem Amanze, Abosede Itunuoluwa Oni, Susan Grant, Matthew Iyobhebhe, Tobiloba Christiana Elebiyo, Damilare Rotimi, Nnaemeka Tobechukwu Asogwa, Babatunji Emmanuel Oyinloye, Basiru Olaitan Ajiboye, Adebola Busola Ojo

**Affiliations:** 1 Phytomedicine, Molecular Toxicology and Computational Biology Research Group, Department of Biochemistry, Landmark University, Omu-Aran, Nigeria; 2 Central Research Laboratory 123B, University Road, Tanke, Ilorin, Nigeria; 3 Department of Biochemistry, Afe Babalola University Ado-Ekiti, Ado-Ekiti, Nigeria; 4 Department of Biochemistry, Federal University Oye-Ekiti, Oye-Ekiti, Nigeria; 5 Department of Biochemistry, Ekiti State University, Ado-Ekiti, Nigeria

**Keywords:** Biochemistry, Molecular biology

## Abstract

The treatment of diabetes involves the use of herbal plants, attracting interest in their cost-effectiveness and efficacy. An aqueous extract of *Persea americana* seeds (AEPAS) was explored in this study as a possible therapeutic agent in rats with diabetes mellitus. The induction of diabetes in the rats was achieved by injecting 65 mg/kg body weight (BWt) of alloxan along with 5% glucose. This study was conducted using thirty-six (36) male Wistar rats. The animals were divided into 6 equal groups, (*n* = 6) and treated for 14 days. In vitro assays for total flavonoid, phenols, FRAP, DPPH, NO, α-amylase, and α-glucosidase, were performed. Biochemical indices fasting blood sugar (FBS), BWt, serum insulin, liver hexokinase, G6P, FBP, liver glycogen, IL-6, TNF-α, and NF-ĸB in the serum, were investigated as well as the mRNA expressions of PCNA, Bcl2, PI3K/Akt in the liver and pancreas. The in vitro analyses showed the potency of AEPAS against free radicals and its enzyme inhibitory potential as compared with the positive controls. AEPAS showed a marked decrease in alloxan-induced increases in FBG, TG, LDL-c, G6P, F-1, 6-BP, MDA, IL-6, TNF-α, and NF-ĸB and increased alloxan-induced decreases in liver glycogen, hexokinase, and HDL-c. The diabetic control group exhibited pancreatic dysfunction as evidenced by a reduction in serum insulin, HOMA-β, expressions of PI3K/AKT, Bcl-2, and PCNA combined with an elevation in HOMA-IR. The HPLC revealed luteolin and myricetin to be the phytochemicals that were present in the highest concentration in AEPAS. The outcome of this research showed that the administration of AEPAS can promote the activation of the PI3K/AkT pathway and the inhibition of β-cell death, which may be the primary mechanism by which AEPAS promotes insulin sensitivity and regulates glycolipid metabolism.

## Introduction

Diabetes mellitus (DM) is among the top 10 reasons for premature death, accounting for over 1,000,000 deaths^1^ worldwide. Though there are two broad categories of diabetes, type-1 diabetes mellitus (T1DM) and type-2 diabetes mellitus (T2DM) which is the most widespread type. T2DM is a public health challenge and has contributed to human morbidity and premature mortality^2^. The prevalence of T2DM is 462 million in 2017 with a worldwide incidence rate of 6059 cases per 100,000 people and is projected to increase to 7079 cases per 100,000 people by 2030^3^. The incidence of T1DM was 15 per 100,000 people and the prevalence was 9.5% (95% CI 0.07 to 0.12) in the world, which was statistically significant^4^.

Diabetes mellitus is a metabolic disorder linked to aberrant glucose concentration in the plasma and leads to insulin deficiency and/or impaired insulin activity. Some factors that affect insulin expression and action are body mass index (BMI), physical inactivity, heavy alcohol consumption, genetic and epigenetics, predisposition, and tobacco smoking^4^.

The characteristic feature of T1DM is often associated with autoimmune destruction of the pancreatic ß-cell, causing total insulin deficiency. The classic symptoms include weight loss, polyuria, polydipsia, polyphagia, and ketoacidosis. Pancreatic ß-cells are essential for maintaining a balance in blood glucose levels by synthesizing and secreting insulin^5^. Prolonged hyperglycemia results in the apoptotic death of pancreatic ß-cells, thus reducing the pancreatic ß-cell volume and stimulating aberrant insulin release and glucose uptake^6^. Hyperglycemia also stimulates the heightened generation of reactive species via NADPH oxidase activity, which potentiates proinflammatory biomarkers, such as IL-1β, IL-6, and TNF-α^7^.

Diabetes and its complications, like other diseases, have been linked to free radical generation, with glucose autoxidation being a major source of free radicals in chronic hyperglycemia^7^. Although free radical production is necessary for normal cellular homeostasis and the body's response to pathogens, many diabetes complications are caused by excessive free radical generation and oxidative stress^6^. The oxidative stress and inflammation caused by persistent hyperglycemia are major contributors to these diabetes complications^8^.

PI3K is a kinase that is involved in regulating both the uptake and utilization of cellular glucose. Based on experimental findings, it is now clear that the release and regulation of insulin by pancreatic cells takes place via a PI3K/AKT mediated pathway^9,10^. In addition to stimulating pancreatic β-cell survival by inhibiting FoxO1, it is implicated in lipotoxicity in pancreatic β-cells. Many studies have shown that the overexpression and activation of PI3K/AKT in β-cells stimulate insulin secretion while the overexpression of inactive mutated forms of this kinase in β-cells and a subsequent reduction in PI3k/AKT activity leads to a lack of insulin secretion^10–12^.

*Persea americana* Mill. (avocado) seed extract pulp, and leaves are rich in phytochemicals, vitamins, micronutrients, and antioxidants^13,14^. Research has identified *Persea americana* as a potent hypoglycemic agent^14–16^. However, the mechanism by which *P. americana* works and its hypoglycemic activity have not been explained. Therefore, our study examined the role of *P.* *americana* Mill. seeds in attenuating alloxan-induced diabetes by suppressing oxidative stress, inflammation, and β-cell apoptotic death, and by upregulating glucose uptake by stimulating the PI3K/AKT signaling pathway.

## Results

### HPLC–UV analysis of AEPAS

The outcomes of the HPLC analysis of AEPAS at 254 nm (Fig. S1), as shown by the chromatograms obtained at different retention times, showed the presence of several constituents, which are ascorbic acid, myricetin, luteolin, and gallic acid (Table 1).

**Table 1:** Bioactive compounds identified in AEPAS.

Compounds	Retention time	Concentration (µg/10 g)
Ascorbic acid	0.915	0.0879
Myricetin	1.265	42.2598
Luteolin	1.423	55.6221
Gallic acid	2.982	1.7266

AEPAS: aqueous extract of *Persea americana* seeds.

### In vitro antioxidant activity of AEPAS

**Figure 1:**
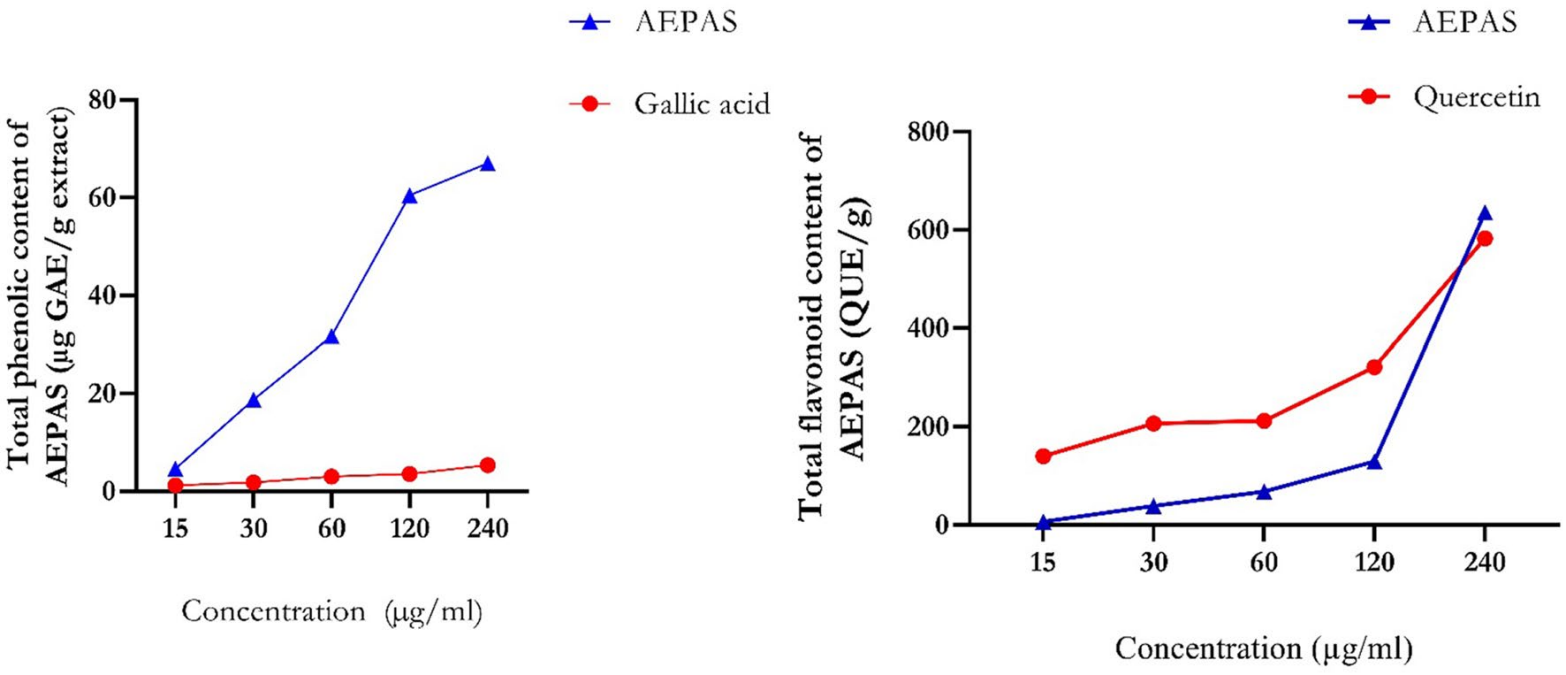
Total phenolic and flavonoid contents of aqueous extract of *P. americana* seeds. Data are expressed as mean ± SEM of duplicate determinations. AEPAS: aqueous extract of Persea americana seeds; GAE: gallic acid; QUE: quercetin.

**Figure 2:**
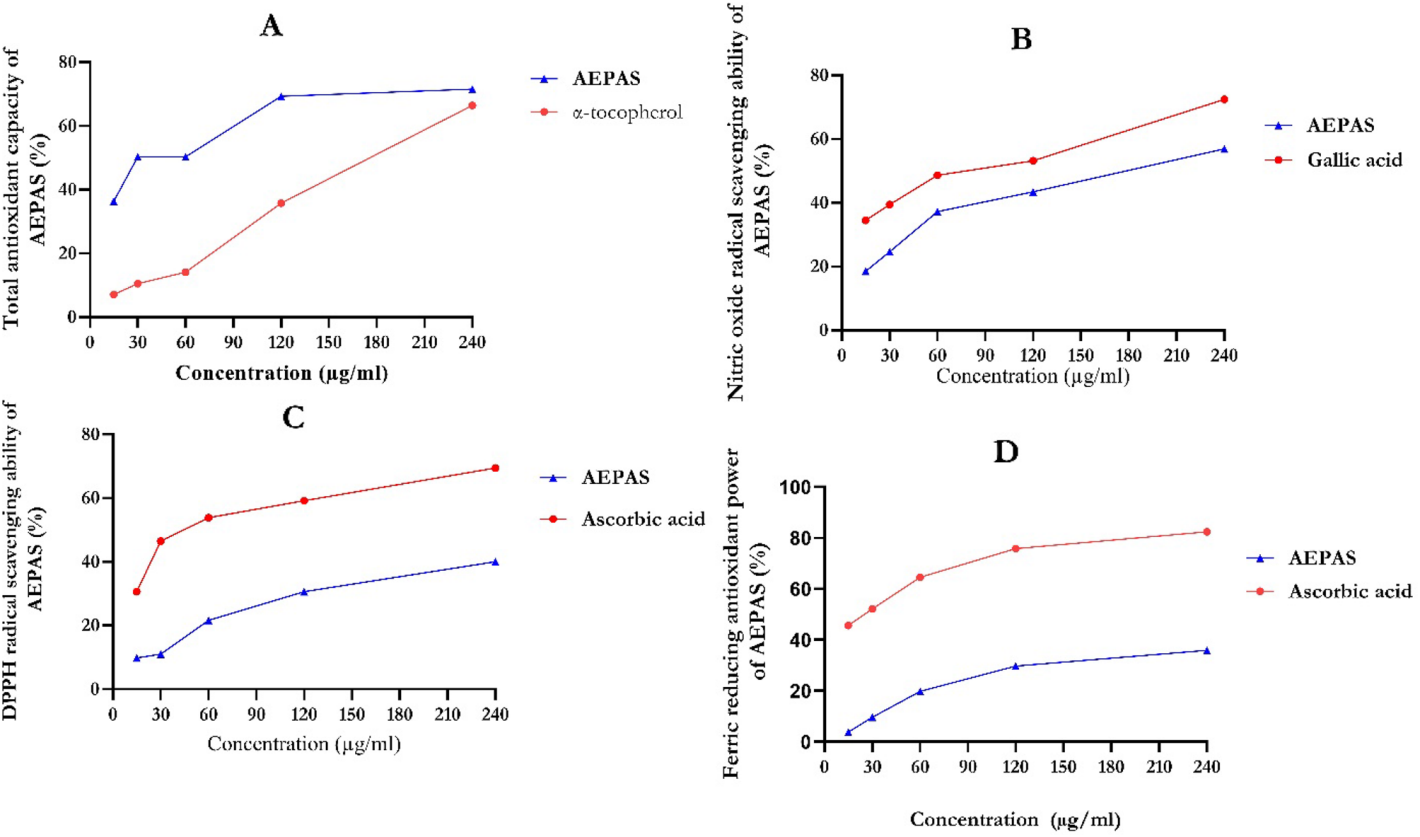
In vitro antioxidant activity of aqueous extract of *P. americana* seeds (**A**) total antioxidant capacity, (**B**) nitric oxide, (**C**) DPPH, and (**D**) ferric reducing ability. Data are expressed as mean ± SEM of duplicate determinations. AEPAS: aqueous extract of *Persea americana* seeds.

As shown in Fig. 1**,** we compared the phenol and flavonoid contents of the AEPAS with standard gallic acid and quercetin. The graph shows the total phenolic content in the sample and the standard, which increases with concentration. The total flavonoid content was concentration dependent as the results showed an increase in flavonoid content with increased concentration. Figure 2 shows an increase in the total antioxidant capacity of AEPAS at a higher concentration. The total antioxidant ability of the extract was greater than α-tocopherol, which was used as the standard. The trends observed in the samples and standards were similar, as both showed increased antioxidant capacity at higher concentrations. Increasing the concentration increased the nitric oxide scavenging capability of AEPAS and gallic acid. We illustrate this in Fig. 2, which shows that the DPPH scavenging ability of AEPAS increases at higher concentrations with a concentration-dependent increase. We observed a similar trend in DPPH activity for standard vitamin C, as shown in Fig. 2. Figure 2 also shows that the reducing power of AEPAS depends on the concentration in Fig. 2.

### α-glucosidase and α-amylase inhibitory activities of AEPAS

**Figure 3:**
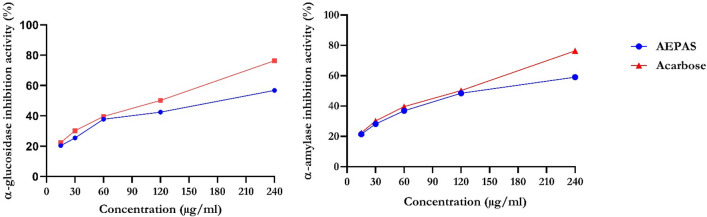
α-Glucosidase and α-amylase inhibitory activities of aqueous extract of *P. americana* seeds. Data are expressed as mean ± SEM of duplicate determinations. AEPAS: aqueous extract of *Persea americana* seeds.

As shown in Fig. 3, acarbose, as a reference drug, had a maximal inhibitory activity of α-glucosidase compared to aqueous seed extract. AEPAS showed its maximal α-glucosidase inhibitory activity at 56.41% and acarbose at 76.41%. In addition, Fig. 3 shows the percent α-amylase inhibitory activities of AEPAS and acarbose. The α**-**amylase inhibitory property exhibited in the aqueous extract was 21.42% compared to 76.41% for acarbose.

### Fasting blood glucose (FBG) level of diabetic rats administered AEPAS

The FBG level of the male rats was elevated 72 h after induction of alloxan to values above 250 mg/dl, which were maintained in the untreated group for the duration of the experiment. A decline in FBG levels was observed after the administration of AEPAS at 26.7 mg/kg, 53.3 mg/kg, and 106.6 mg/kg BWt (Table 2). The reduction in the FBG level in the animal groups treated with 53.3 mg/kg and 106.6 mg/kg BWt yielded values in alignment with the nondiabetic group (Table 2). We found the most profound effect at 106.6 mg/kg BWt.

**Table 2:** Fasting blood glucose levels of alloxan-induced diabetic rats before and after oral administration of aqueous extract of *P. americana* seeds.

Treatment groups	Before alloxan administration	After 48 h of alloxan administration	After 7 days of *P. americana* extract administration	After 14 days of *P. americana* extract administration
Normal control	69.91 ± 1.73	69.91 ± 1.73	69.91 ± 1.73	79.92 ± 1.94
Diabetic control	67.68 ± 2.88	365.38 ± 64.45^#^	395.82 ± 16.92^#^	444.96 ± 38.57^#^
Diabetic + metformin	53.10 ± 0.95	398.70 ± 72.23^#^	325.35 ± 61.22^#^	88.38 ± 3.12*
Diabetic + AEPAS (26.7 mg/kg)	57.24 ± 4.17	450.90 ± 36.59^#^	217.22 ± 2.14**	118.22 ± 14.34*
Diabetic + AEPAS (53.3 mg/kg)	40.32 ± 5.10	376.92 ± 15.30^#^	216.36 ± 27.92**^#^	88.56 ± 6.11*
Diabetic + AEPAS (106.6 mg/kg)	62.37 ± 4.27	290.78 ± 59.89^#^	99.99 ± 7.79*	85.68 ± 4.36*

Data are expressed as mean ± SEM (*n* = 6). *Statistically significant (*p* < 0.05) to DC; ^#^Statistically significant (*p* < 0.05) to NC; **P < 0.01 is considered as very significant.

AEPAS: Aqueous extract of *P. americana* seeds.

### Body weight of diabetic rats administered AEPAS

We witnessed a notable decrease in the body weight in the animals, apparently resulting from the administration of alloxan (Table 3), as evidenced by the weight gain in the normal control group. Treatment with AEPAS at 26.7 mg/kg, 53.3 mg/kg, and 106.6 mg/kg BWt resulted in slight increases in the BWt of the rats (Table 3) with the increases being dose-dependent.

**Table 3:** Body weight of alloxan-induced diabetic rats before and after oral administration of aqueous extract of *P. americana* seeds.

Treatment groups	Initial weight (g)	Final weight (g)	% weight change
Normal control	243.57 ± 11.12	255.46 ± 7.08	11.89↑
Diabetic control	242.06 ± 14.79*	188.05 ± 24.28^#^	− 54.01↓
Diabetic + metformin	245.33 ± 9.42*	210.27 ± 11.84*	− 35.06↓
Diabetic + AEPAS (26.7 mg/kg)	252.72 ± 2.69*	240.37 ± 2.82*	− 12.35↓
Diabetic + AEPAS (53.3 mg/kg)	247.55 ± 2.33*	254.50 ± 0.83*	6.95↑
Diabetic + AEPAS (106.6 mg/kg)	254.75 ± 1.98*	274.01 ± 6.74*	19.26↑

Data are expressed as mean ± SEM (*n* = 6). *Statistically significant (*p* < 0.05) to DC; ^#^Statistically significant (*p* < 0.05) to NC.

AEPAS: Aqueous extract of *P. americana* seeds; Weight loss (↓); Weight gain (↑).

### Serum insulin levels, HOMA-IR and HOMA- β levels of diabetic rats administered AEPAS

From the results presented in Table 4, the serum insulin and HOMA-β levels of the diabetic untreated group showed a remarkable decrease whereas the HOMA-IR levels increased. Administration of AEPAS at 26.7 mg/kg, 53.3 mg/kg, and 106.6 mg/kg resulted in elevated serum insulin and HOMA-β levels with a noteworthy decrease in the HOMA-IR levels.

**Table 4:** Serum insulin levels and HOMA-IR and HOMA-β scores of alloxan-induced diabetic rats after oral administration of aqueous extract of *P. americana* seeds.

Groups	Parameters		
	Insulin (U/l)	HOMA-IR	HOMA-β
Normal control	10.55 ± 0.03	2.03 ± 0.07	268.81 ± 45.62
Diabetic control	5.74 ± 0.40^#^	7.07 ± 0.38^#^	4.75 ± 0.48^#^
Diabetic + metformin	7.49 ± 0.05*	1.55 ± 0.04*	133.05 ± 18.44*
Diabetic + AEPAS (26.7 mg/kg)	9.30 ± 0.06*	2.27 ± 0.29*	150.72 ± 81.47*
Diabetic + AEPAS (53.3 mg/kg)	9.55 ± 0.19*	1.85 ± 0.06*	248.01 ± 54.98*
Diabetic + AEPAS (106.6 mg/kg)	10.40 ± 0.14*	2.48 ± 0.17*	120.71 ± 25.87*

Data are expressed as mean ± SEM (*n* = 6). *Statistically significant (*p* < 0.05) to DC; ^#^Statistically significant (*p* < 0.05) to NC.

AEPAS: Aqueous extract of *P. americana* seeds; HOMA-IR: homeostatic model assessment of insulin resistance: [(Fasting serum insulin in U/l *fasting blood glucose in mmol/l)/22.5]; HOMA-β: homeostatic model assessment of β-cell function: [(Fasting serum insulin in U/l *20/fasting blood glucose in mmol/l-3.5)]; conversion factor: insulin (1U/l = 7.174 pmol/l).

### Antioxidant markers in experimental DM

The activities of glutathione peroxidase (GPx), glutathione-S-transferase (GST), reduced glutathione (GSH), catalase (CAT), and superoxide dismutase (SOD) in the liver were in contrast to the untreated diabetic rats when either AEPAS or metformin was administered to diabetic rats. Lipid peroxidation (MDA) levels decreased after administration of either metformin or AEPAS, in contrast to the elevated levels in the untreated diabetic rats, and the results were dose-dependent (Table 5). The activities of GPx, GST, GSH, CAT, and SOD in the pancreas were elevated and the MDA levels decreased after administration of AEPAS and metformin compared with the diabetic untreated rats (Table 6).

**Table 5:** Hepatic antioxidant markers of alloxan-induced diabetic rats after oral administration of aqueous extract of *P. americana* seeds.

Groups	Parameters					
	MDA (nmol/l)	CAT (U/mg protein)	SOD (U/mg protein)	GPX (U/mg protein)	GSH (µmol/mg tissue)	GST (U/mg protein)
Normal control	2.56 ± 0.63	10.22 ± 0.03	8.14 ± 0.01	17.12 ± 0.02	90.08 ± 11.71	11.09 ± 0.24
Diabetic control	6.11 ± 0.66^#^	1.55 ± 0.03^#^	0.56 ± 0.03^#^	4.56 ± 0.34^#^	42.27 ± 5.43^#^	0.25 ± 0.02^#^
Diabetic + metformin	2.49 ± 0.56*	8.12 ± 0.46*	6.14 ± 0.07*	10.99 ± 0.69*	86.33 ± 9.08*	9.78 ± 0.13*
Diabetic + AEPAS (26.7 mg/kg)	3.22 ± 0.54*	5.56 ± 0.09*	4.07 ± 0.02*	7.75 ± 0.14*	73.23 ± 9.08*	6.44 ± 0.09*
Diabetic + AEPAS (53.3 mg/kg)	2.63 ± 0.66*	6.77 ± 0.25*	5.09 ± 0.01*	8.83 ± 0.03*	91.87 ± 9.72*	7.54 ± 0.06*
Diabetic + AEPAS (106.6 mg/kg)	2.02 ± 0.55*	9.15 ± 0.48*	7.15 ± 0.02*	10.13 ± 0.19*	107.43 ± 7.63*	10.03 ± 0.13*

Data are expressed as mean ± SEM (n = 6). *Statistically significant (*p* < 0.05) to DC; ^#^Statistically significant (*p* < 0.05) to NC.

AEPAS: aqueous extract of *P. americana* seeds; MDA: malondialdehyde; CAT: catalase; SOD: superoxide dismutase; GPX: glutathione peroxidase; GSH: reduced glutathione; GST: glutathione s-transferase.

**Table 6:** Pancreatic antioxidant markers of alloxan-induced diabetic rats after oral administration of aqueous extract of *P. americana* seeds.

Groups	Parameters					
	MDA (nmol/l)	CAT (U/mg protein)	SOD (U/mg protein)	GPX (U/mg protein)	GSH (µmol/mg tissue)	GST (U/mg protein)
Normal control	1.66 ± 0.14	10.97 ± 0.01	6.34 ± 0.03	2.16 ± 0.02	8.12 ± 0.11	9.38 ± 0.52
Diabetic control	7.75 ± 0.12^#^	1.16 ± 0.01^#^	0.12 ± 0.02^#^	0.02 ± 0.03^#^	3.12 ± 0.26^#^	0.16 ± 0.02^#^
Diabetic + metformin	1.68 ± 0.58*	8.82 ± 0.03*	5.35 ± 0.03*	1.45 ± 0.01*	7.52 ± 0.29*	8.76 ± 0.29*
Diabetic + AEPAS (26.7 mg/kg)	3.56 ± 0.59*	4.46 ± 0.03*	3.22 ± 0.03*	1.23 ± 0.03*	5.69 ± 0.33*	6.37 ± 0.19*
Diabetic + AEPAS (53.3 mg/kg)	2.85 ± 0.27*	6.80 ± 0.03*	4.31 ± 0.03*	1.38 ± 0.01*	6.46 ± 0.25*	7.57 ± 0.21*
Diabetic + AEPAS (106.6 mg/kg)	1.42 ± 0.13*	8.16 ± 0.06*	5.36 ± 0.06*	1.98 ± 0.01*	9.38 ± 0.52*	8.11 ± 0.39*

Data are expressed as mean ± SEM (*n* = 6). *Statistically significant (*p* < 0.05) to DC; ^#^Statistically significant (*p* < 0.05) to NC.

AEPAS: aqueous extract of *P. americana* seeds; CAT: catalase; SOD: superoxide dismutase; GPX: glutathione peroxidase; GSH: reduced glutathione; GST glutathione s-transferase; MDA: malondialdehyde.

### Serum lipid parameters in experimental DM administered AEPAS

Administration of alloxan led to an increased concentrations of VLDL-c, LDL-c, TG, CRI, AI, and total cholesterol with a reduction in HDL-c levels (Table 7). Administration of the various dosages of AEPAS at 26.7 mg/kg, 53.3 mg/kg, and 106.6 mg/kg resulted in a decrease in triglycerides, LDL-c, total cholesterol, CRI, VLDL-c, and AI levels in contrast to the diabetes-induced rats. The doses of the extract elevated the serum HDL-c levels when compared to the diabetic rats. Metformin treatment resulted in a significant decrease in triglycerides, LDL-c, total cholesterol, CRI, VLDL-c, and AI levels with a corresponding improvement in HDL-c levels.

**Table 7:** Lipid profile of alloxan-induced diabetic rats after oral administration of aqueous extract of *P. americana* seeds.

Groups	Parameters						
	TC (mmol/l)	HDL-c (mmol/l)	TG (mmol/l)	VLDL-c (mmol/l)	LDL-c (mmol/l)	AI	CRI
Normal control	38.63 ± 2.53	30.11 ± 3.27	27.97 ± 1.29	5.59 ± 0.26	2.92 ± 1.81	0.22 ± 0.04	1.30 ± 0.07
Diabetic control	103.97 ± 4.33^#^	6.74 ± 0.27^#^	73.63 ± 0.15^#^	14.73 ± 0.03^#^	82.50 ± 4.03^#^	0.94 ± 0.00^#^	15.42 ± 0.02^#^
Diabetic + metformin	66.02 ± 5.07*	21.07 ± 0.39*	63.85 ± 0.30*	12.77 ± 0.06*	32.18 ± 5.25*	0.68 ± 0.03*	3.14 ± 0.27*
Diabetic + AEPAS (26.7 mg/kg)	49.17 ± 0.32*	24.27 ± 0.18*	53.21 ± 0.12*	10.64 ± 0.02*	14.26 ± 0.47*	0.51 ± 0.01*	2.03 ± 0.03*
Diabetic + AEPAS (53.3 mg/kg)	44.43 ± 0.92*	26.09 ± 0.03*	42.96 ± 0.04*	8.59 ± 0.01*	9.74 ± 0.94*	0.41 ± 0.01*	1.70 ± 0.04*
Diabetic + AEPAS (106.6 mg/kg)	38.52 ± 0.50*	28.80 ± 0.21*	34.96 ± 2.15*	6.99 ± 0.43*	2.73 ± 0.83*	0.25 ± 0.01*	1.34 ± 0.03*

Data are expressed as mean ± SEM (*n* = 6). *Statistically significant (*p* < 0.05) to DC; ^#^Statistically significant (*p* < 0.05) to NC.

AEPAS: Aqueous extract of *P. americana* seeds; TC: total cholesterol; TG: triglyceride; HDL-c: high density lipoprotein-cholesterol; AI: atherogenic index: [(TC-HDL-c)/HDL-c]; CRI: coronary index: [(TC(mg/dl)/HDL-c(mg/dl)]; VLDL-c: very low density lipoprotein-cholesterol: [TG/5]; LDL-c: low density lipoprotein-cholesterol: [TC-HDL-(TG/5)].

### Hepatic glycogen and carbohydrate metabolizing enzymes in diabetic rats administered AEPAS

The levels of hepatic glycogen and the glycolytic enzyme, hexokinase, were diminished in the diabetic rats, and we observed a remarkable increase of these in the treatment groups after administration of either metformin or AEPAS (Table 8). We found that the activities of the gluconeogenesis enzymes G6Pase and F-1,6-BPase increased in the diabetic control group, but administration of either AEPAS or metformin yielded a notable decrease in the treatment groups (Table 8).

**Table 8:** Hepatic glycogen and carbohydrate metabolizing enzyme levels after oral administration of aqueous extract of *P. americana* seeds.

Treatment groups	Hepatic glycogen^α^	Hexokinase^β^	Fructose-1,6-bisphosphatase^γ^	Glucose-6-phosphatase^γ^
Normal control	53.20 ± 1.15	1.52 ± 0.50	1.28 ± 0.04	22.61 ± 1.42
Diabetic control	15.84 ± 3.50^#^	0.26 ± 0.04^#^	3.21 ± 0.32^#^	94.73 ± 2.47^#^
Diabetic + metformin	55.98 ± 2.16*	1.11 ± 0.07*	0.87 ± 0.03*	60.88 ± 2.31*
Diabetic + AEPAS (26.7 mg/kg)	54.84 ± 0.86*	0.76 ± 0.13*	0.94 ± 0.07*	53.98 ± 1.87*
Diabetic + AEPAS (53.3 mg/kg)	55.27 ± 0.57*	1.14 ± 0.07*	0.99 ± 0.11*	47.36 ± 1.32*
Diabetic + AEPAS (106.6 mg/kg)	92.05 ± 3.93*	1.31 ± 0.07*	1.13 ± 0.10*	36.90 ± 3.76*

Data are expressed as mean ± SEM (*n* = 6). *Statistically significant (*p* < 0.05) to DC; ^#^Statistically significant (*p* < 0.05) to NC.

AEPAS: Aqueous extract of *P. americana* seeds; ^α^: Unit for glycogen (mg of glucose/g of wet tissue); ^β^: Unit for hexokinase (µmole glucose-6-phosphate formed/min/mg protein); ^γ^: Unit for fructose-1,6-bisphosphatase and glucose-6-phosphatase (µmole phosphate liberated/min/mg protein).

### Pro- and anti-inflammatory markers of diabetic rats administered AEPAS

**Figure 4:**
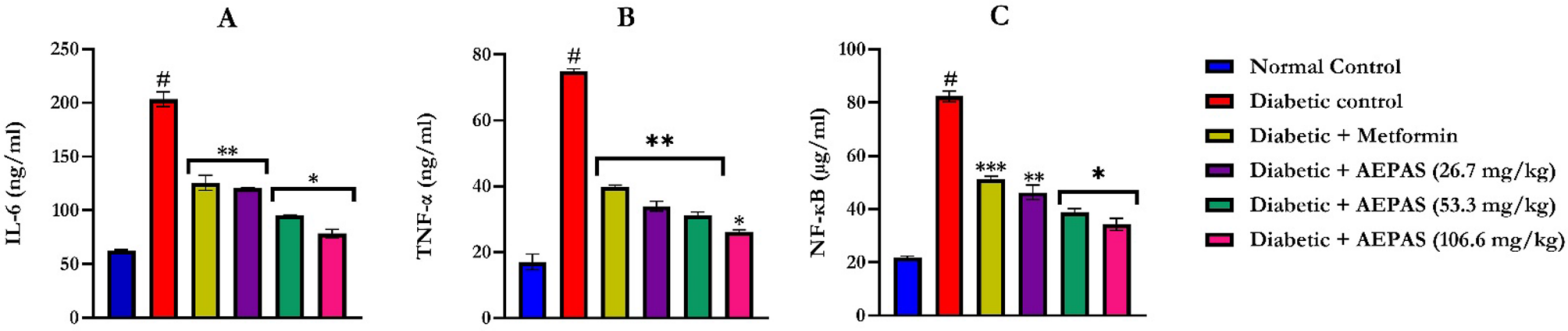
Plasma pro- and anti-inflammatory markers of diabetic rats administered AEPAS, (**A**) interleukin-6, (**B**) tumor necrosis factor-α*,* and (**C**) nuclear factor-kappa B of alloxan-induced diabetic rats orally administered AEPAS. Data are expressed as mean ± SEM (*n* = 6); AEPAS: aqueous extract of *Persea americana* seeds; IL-6: Interleukin-6; TNF-α: Tumor necrosis factor-alpha; NF-κB: Nuclear factor-kappa B; **#**: significantly different from normal control (*P* < .05); *, **,*** is significantly different from diabetic control at *P* < 0.05, < 0.01, < 0.001).

Administration of alloxan increased the levels of IL-6, TNF-α, and NF-κB (Fig. 4) in the plasma of the rats (*p* < 0.05) compared with those of the control rats. AEPAS reduced the levels of IL-6, TNF-α, and NF-κB (p < 0.05) compared to the diabetic rats, as did treatment with metformin. AEPAS at 26.7, 53.3, and 106.6 mg/kg BWt showed the reversal of the alloxan treatment-related rises in the concentrations of IL6, TNF-α, and NF-κB (Fig. 4).

### Gene expressions of PI3K, AKT, and apoptotic markers of diabetic rats administered AEPAS

**Figure 5:**
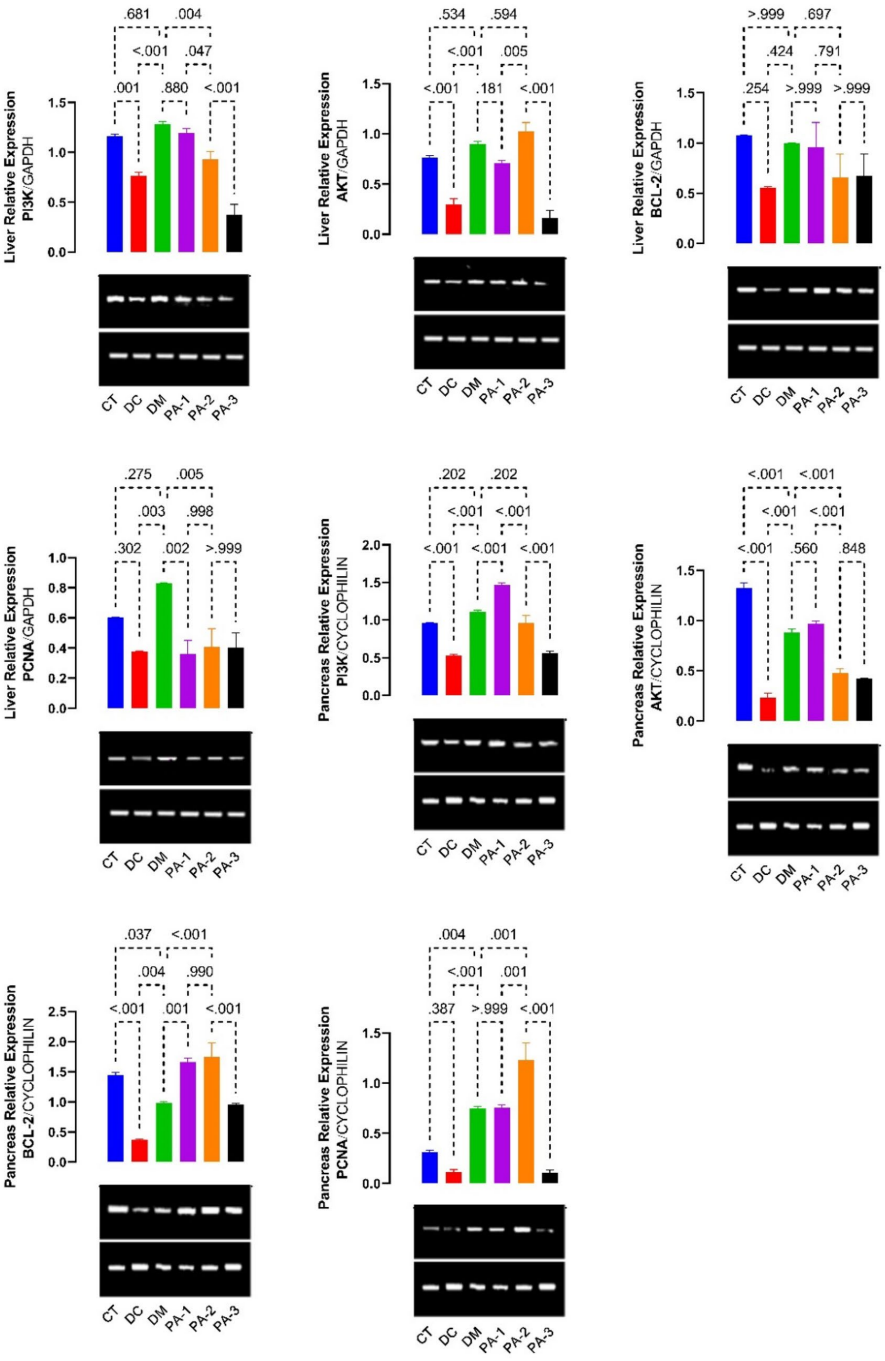
Effect of AEPAS on PI3K, AKT, Bcl2, and PCNA in the liver and pancreas of diabetic rats. AEPAS: aqueous extract of *P*. *americana* seeds; PCNA: proliferating cell nuclear antigen; CT: control group; DC: diabetic control; DM: diabetic + metformin group; PA-1: *P. americana* treated group (26.7 mg/kg); PA-2: *P. americana* treated group (53.3 mg/kg) PA-3: *P. americana* treated group (106.6 mg/kg).

The PI3K, AKT, Bcl-2, and PCNA mRNA expression levels in the liver and pancreas are shown in Fig. 5. The the mRNA expression levels of PI3K, AKT, Bcl-2, and PCNA were down-regulated in the liver and the pancreas of the diabetic rats (Fig. 5). Treatment with 26.7 or 53.3 mg/kg AEPAS and metformin raised the mRNA expression levels of PI3K and AKT in the liver, while AEPAS at all doses increased the expression of Bcl-2 and PCNA in the liver. In addition, AEPAS at all doses and metformin caused an elevation in the mRNA expression of PI3K, AKT, and Bcl-2 in the pancreas. In contrast, the PCNA mRNA expression at 26.7 and 53.3 mg/kg increased in the diabetic rats but decreased at 106.6 mg/kg AEPAS in the pancreas.

### Histological study of pancreatic tissues

**Figure 6:**
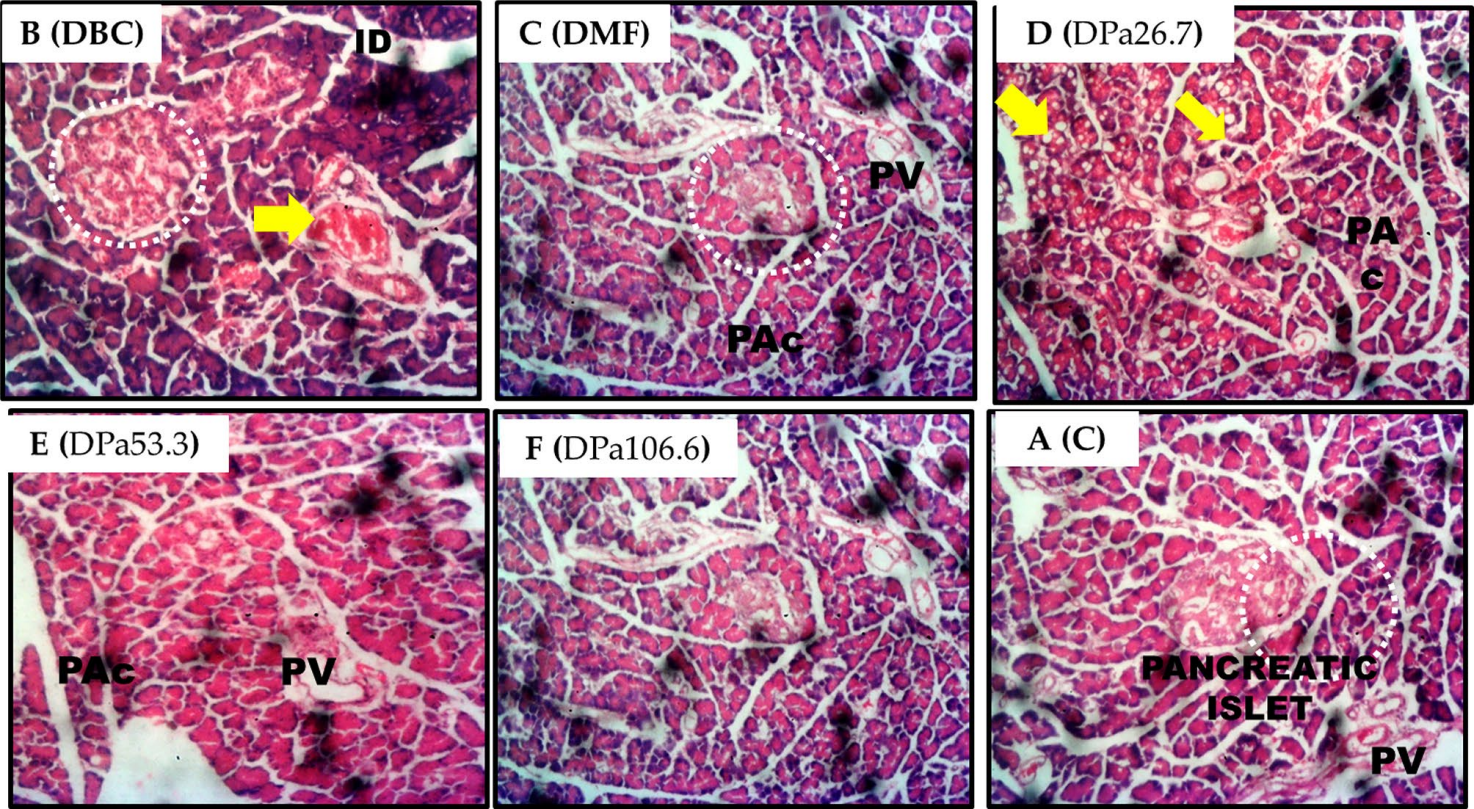
Cross section of the pancreas of rats after administration of aqueous extract of *P*. *americana* seeds. (**A**) NC, Normal control; (**B**) DBC, Diabetic Control; (**C**) DMF, Diabetic Metformin; (**D**) DPa26.7: Diabetic *P. americana* aqueous seed extract (26.7 mg/kg body weight); (**E**) DPa53.3: Diabetic *P. americana* aqueous seed extract (53.3 mg/kg body weight); (**F**) DPa106.6: Diabetic *P. americana* aqueous seed extract (106.6 mg/kg body weight).

Alloxan induction resulted in the partial destruction of the β-cells of the pancreatic islets when compared to control rats (Fig. 6). Treatment with AEPAS, on the other hand, improves and restores the damaged pancreatic islets cells at all doses.

## Discussion

Researchers have related the antidiabetic properties of several medicinal plants to bioactive compounds such as phenolics, flavonoids, and tannins^17^. Over the years, scientists have explored these chemical compounds in managing several diseases by folklore medicine^18–20^. The HPLC of AEPAS confirmed the presence of ascorbic acid, myricetin, luteolin, and gallic acid. Ascorbic acid is available as a natural antioxidant in some systems. Advanced glycation end products, glycosylation of proteins, the polyol pathway, and auto-oxidation of glucose are involved in the pathogenesis of T1DM and T2DM. Thus, one important defense against such impairment is antioxidant compounds such as ascorbic acid^21^.

Myricetin, a flavonoid compound, was documented to have antioxidant and antidiabetic activities. Myricetin facilitated the metabolic action of insulin by stimulating phosphatidylinositol 3-kinase (PI3K) and its effectors^22^.

Luteolin is an active flavonoid with an extensive array of pharmacological properties. The well-known pharmacological properties of luteolin, for example, its antioxidant, anti-inflammatory, anti-apoptotic, and antidiabetic properties, have been demonstrated^23^.

The antioxidant property and inhibitory activities of gallic acid on marker enzymes (α-glucosidase and α-amylase) linked to diabetes mellitus have been shown by Mackensie et al.^24^, and improvement in IR sensitivity by gallic acid may account for its anti-diabetic property^25^. All the activities of ascorbic acid, myricetin, luteolin, and gallic acid summarized above could have accounted for the antioxidant and antidiabetic properties we observed for AEPAS.

Recently, phenolic compounds have attracted considerable attention for their promising use as novel nutraceuticals or biological products because of their notable antiradical and anti-inflammatory properties, which may be concentration dependent^26^. Flavonoids are a major group of secondary metabolites, and many experiments have reported their biological properties^27^. This study showed that the total phenolic and flavonoid content of AEPAS operated in a concentration-dependent manner.

Free radicals have been linked to diseases such as T2DM^28^. Researchers documented that hyperglycemia produces free radicals that turn on the free radical/antioxidant defense system, producing oxidative stress. We can evaluate the antioxidant activities of therapeutic plants via their total antioxidant capacity and their reducing and radical scavenging properties^29^. Recent studies reported that strong antioxidants present in therapeutic foods and plants can be useful in neutralizing the effects of stress in diseases such as type-2 diabetes^30^. The efficiency of AEPAS as an antioxidant could be due to phenolics and flavonoids, a conclusion that is further supported by the ability of AEPAS to reduce ferric ions and to neutralize NO and DPPH radicals. From this study, AEPAS revealed a high total antioxidant capacity, reducing capacity, and scavenging power. An overabundance of nitric oxide can lead to tissue injury and Type-2 diabetes-linked cardiovascular problems^31^. Hence, AEPAS could inhibit nitric oxide radicals in a concentration-dependent manner. This could be because of the compounds, including ascorbic acid, myricetin, luteolin, and gallic acid, present in AEPAS, which have been reported to have these properties.

The treatment of hyperglycemia is the essential goal for managing diabetes mellitus. One of the most significant beneficial methods is reducing postprandial hyperglycemia by hindering carbohydrate absorption and digestion^32^. Anti-diabetic agents function by inhibiting α-amylase and α-glucosidase enzymes relevant to T2DM^33^. These enzymes perform a key role in the breakdown of nutritional carbohydrates to glucose^33^. The inhibitory activity of the AEPAS on α-amylase and α-glucosidase indicates its potential as an antidiabetic treatment. The inhibition of these enzymes by AEPAS clarifies its capability to delay the breakdown of carbohydrates, hence controlling the FBG level. We can credit these activities to the metabolites, phenolics, and flavonoids that have been described as components of herbal extracts with anti-diabetic properties^34^. It is important to note that the α-amylase activity of AEPAS compared with that of acarbose, whose mechanism of action involves inhibiting these enzymes.

Alloxan-induced diabetes is a valuable experimental model for examining the antidiabetic properties of many agents^35^. The mechanism by which alloxan induces diabetes is that it selectively inhibits glucose-induced insulin production via a specific hexokinase inhibition that triggers a condition similar to T2DM via its capacity to induce ROS, resulting in pancreatic β-cell toxicity^36^. The reference antidiabetic agent, metformin, acts in many ways, including decreasing glucose generation, improving lipid oxidation in liver cells, and/or increasing the uptake of glucose^36^. The increased FGB level observed after 72 h of alloxan administration supports the idea that glucose-induced insulin production is inhibited through specific hexokinase inhibition and selective toxicity on the β-cells^36^. The decreased level of serum FBG in both the AEPAS and metformin-treated groups compared with the diabetic group supports the view that the therapeutic properties displayed by AEPAS could result from phenolics components such as gallic acid and myricetin. The reduction in serum glucose level by the AEPAS could be due to the increased uptake of glucose evidenced by the improved glycolytic pathway in this study.

A severe loss in bodyweight typifies alloxan-induced diabetes^37^. The difference in energy consumption and usage leads to an alteration in body weight^37^. The decrease in body weight in diabetic rats might be the result of a decrease in glucose metabolism, elevated metabolism of fats, or the structural breakdown of proteins that provide an alternate source of energy^37^. The increased body weight of the diabetic rats treated with AEPAS at the highest dose could have resulted from greater glycemic control through improved insulin secretion. This antidiabetic property indicates that AEPAS may promote insulin production from the residual β-cells or restored β-cells which could activate the hormones involved in fat storage.

Insulin resistance and pancreatic β-cell dysfunction typifies diabetes mellitus^38^. The quantitative assessment of insulin resistance (HOMA-IR) and insulin production/β-cell function (HOMA-β) are vital keys for measuring IR and assessing β-cell function^38^. Improved insulin production and insulin sensitivity in response to AEPAS showed its ability to improve HOMA-IR and HOMA-β. It would be reasonable to credit this effect to the flavonoids in AEPAS since they have been shown to promote the restoration of pancreatic β-cells and increase insulin release in diabetes-induced rats^38^. This corresponds to the findings of a previous study from our laboratory.

Enzymatic antioxidants (CAT, SOD, GPx, GSH, and GST) and non-enzymatic antioxidants (MDA) perform a crucial function in maintaining the biological levels of oxygen and hydrogen peroxide via improving the dismutation of O_2_ radicals and destroying organic peroxides produced from alloxan exposure^39^. This current study showed that alloxan-induced diabetes unbalanced the activity of liver marker enzymes. The observed reduction in the activities of CAT, SOD, GPx, and GST and the levels of GSH in the liver and pancreas of diabetic rats may be due to the chemical reduction of alloxan to dialuric acid to produce redox intermediates and of oxidized glutathione (GSSG) to produce radicals, such as the OH radical that is the main toxic ROS species^36^. This may account for the inadequacy of the defense system in alleviating ROS facilitated injury^40^. The improved activities of these enzymes by AEPAS appears to have reduced the difference between the production of ROS and enzymatic antioxidant activities in the diabetic rats. Also, the formation of ROS is prevented by the destruction of radicals by these antioxidant enzymes. Thus, the ability of AEPAS to attenuate the changed antioxidant enzymes in alloxan-induced diabetic rats indicates its radical scavenging property. This may be due to the phenolics and flavonoids in AEPAS, which have been identified as having radical scavenging effects^27^.

Dyslipidemia, increments in the concentrations of TC, TG, and alterations in lipoprotein components, is a recognized challenge in diabetic patients^41,42^. Also, the high concentrations of LDL-c and AI induced by alloxan imply a propensity for cardiovascular issues^42^. The increased levels of TC in diabetic animals are due to the inactivation of lipoprotein lipase^41,42^. The ability of AEPAS to restore the deranged metabolic pathways could be the result of lipase inhibition via insulin and a subsequent reduction in the rate of lipid degradation, biotransformation of free fatty acids to phospholipids. This effect by AEPAS might result from the presence of flavonoids, which have been reported to reduce lipid metabolism^42^.

Glycogen concentrations in tissues such as the liver are a direct indication of insulin action in that insulin improves internal glycogen deposition by inhibiting glycogen phosphorylase^43^. Depletion of glycogen by alloxan administration could result from a decrease in the activity of hexokinase. The restoration of hepatic glycogen after the administration of AEPAS may have resulted from its ability to improve insulin release from the pancreas^43^. However, this could also have been because of polyphenols, which have been reported to have insulin mimetic activity thus, giving rise to direct peripheral glucose uptake^43^.

The liver is a significant organ that performs a vital function in the glycolytic and gluconeogenic pathways. Hexokinase, fructose-1,6-bisphosphatase, and glucose-6-phosphatase are key enzymes in glucose metabolism^44^. The decrease in hexokinase activity in diabetic animals could be because of reduced glycolysis and diminished use of glucose for energy creation. Increased activity resulting from AEPAS appears to have enhanced the use of glucose for energy creation, which suggests a higher glucose uptake from the blood by hepatocytes and improved glycolysis.

The activity of these enzymes (F-1,6-BPase and G-6-Pase may be elevated in diabetes^45^. The increase in F-1,6-BPase and G-6-Pase in the hepatic tissue of diabetic rats may be related to insulin inadequacy and overproduction of glucose^46^. In addition, the decrease in F-1,6-BPase and G-6-Pase after AEPAS treatment could have resulted from inhibiting gluconeogenesis through improved insulin production, modulating the activity of F-1,6-BPase and G-6-Pase via the control of cyclic adenosine monophosphate (cAMP) or inhibition of glycolysis^47^. The ability of AEPAS to modulate F-1,6-BPase and G-6-Pase could be the result of the action of flavonoids and phenolics, which play a vital role in reversing F-1,6-BPase and G-6-Pase to near normal through enhanced insulin secretion by their antioxidant potential. This further supported the antihyperglycemic activity of the AEPAS.

**Figure 7:**
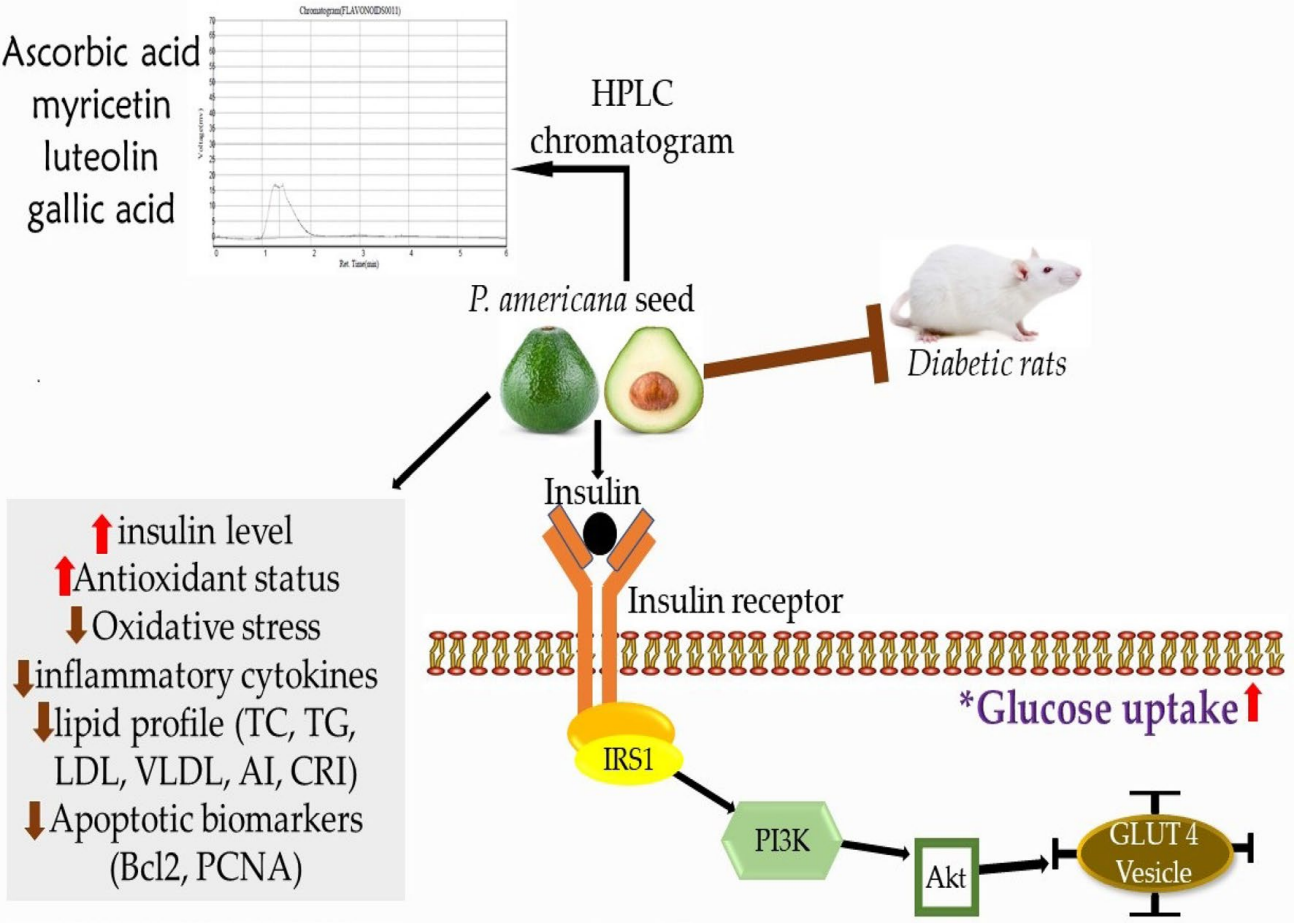
Proposed mechanism of action of *Persea americana* seeds in diabetic rats on improving insulin binding and increasing glucose metabolism. *P. americana*, increases the PI3K/Akt, PCNA, and Bcl2 expression in the insulin signaling pathways. This leads to an increase in insulin sensitivity and a reduction in blood glucose.

The PI3K/AKT signaling pathway is linked to the glucose metabolism essential for insulin stimulated glucose intake in the liver, as earlier documented by Nandipati et al.^48^_,_ and Yu et al.^49^ Akt activation stimulated the cell’s existence by phosphorylation^50^. It facilitated the metabolic action of insulin through the activation of PI3K and its effectors, the protein kinase B (PKB/Akt) kinases. In addition, the AMPK signaling pathway may facilitate the impact of the insulin-independent response to glucose uptake. AEPAS could protect against alloxan-induced damage through an anti-apoptotic effect by increasing the mRNA expression of the phosphorylation of Akt^50^. AEPAS reversed the reduced mRNA expression of PI3K and AKT levels in diabetic rats (Fig. 7).

The antiapoptotic Bcl-2 is a major molecule involved in apoptosis. Alloxan-induced diabetic rats had reduced Bcl-2 expression in the liver and pancreas tissues while treatment with AEPAS altered the balance of the antiapoptotic (Bcl-2) molecules and prevented cell death of the hepatic and pancreatic cells at all doses, as did metformin. The findings from this study (elevated MDA level and reduced SOD activity in the liver and pancreatic tissues of diabetic rats) agree with a study describing the elevated generation of radicals resulting in cell death of hepatic and pancreatic cells^51^. In this case, the apoptotic pathway requires additional examination to determine whether caspase(s) and cytochrome c are involved, as documented in other research experiments^52^.

As a valuable proliferation marker, proliferating cell nuclear antigen (PCNA) expression performs exclusive functions at the start of cell propagation by facilitating DNA polymerase. PCNA also performs key functions in the eukaryotic cell cycle in addition to stimulating the development of antibodies towards foreign compounds^53^. In this study, the mRNA expression of PCNA was up-regulated in the hepatocytes and pancreatic tissues of the normal rats and downregulated in the diabetic rats. AEPAS improved the PCNA expression in the liver and pancreatic tissues of diabetic rats.

In conclusion, AEPAS attenuates insulin resistance in alloxan-induced diabetic rats. The potential molecular mechanisms of AEPAS are an increase in glucose uptake, increased hexokinase activity, increased insulin levels, and a decrease in pancreatic β-cells apoptosis via activation of the PI3K/Akt signaling pathway in diabetic rats (Fig. 7). AEPAS could be an excellent source of antidiabetic agents as it controls the hyperglycemic index and other associated biochemical indices.

## Materials and methods

### Chemicals

Alloxan was purchased from Sigma-Aldrich, Steinheim, Germany. Methanol, and Folin–Ciocalteu’s reagents were both acquired from Merck (Darmstadt, Germany); α-amylase, diphenylamine, acarbose, and α-glucosidase were all products of Sigma-Aldrich, Steinheim, Germany. All chemical agents, as well as standards, were analytical grade, unless otherwise noted.

### Plant material

The seeds of *P. americana* were collected from a local farm settlement in Ado-Ekiti metropolis, Ekiti State, after obtaining permission from the farm owner to collect the seeds of *P. americana* on October 10, 2020. Mr. Odewo at the Forestry Research Institute of Nigeria (FRIN) identified the plant and provided FHI 113162 as the voucher number. The link http://www.theplantlist.org was used to check the name of the plant. Studies complied with local and national ethical guidelines/regulations on the usage of plants.

### Preparation of aqueous extract of *P. americana* seeds (AEPAS)

*Persea americana* seeds were sliced into smaller parts and dried for 4 weeks at 25 °C, after which they were ground into powder using an electric blender (Kenwood, Model BL490, China). 35 g of dried seed was soaked in distilled water for 48 h to obtain the aqueous extracts^17^. We concentrated the aqueous extract obtained using a freeze-dryer (Modulyo Freeze Dryer, Edward, England) to obtain an 18.5 g yield.

### High-performance liquid chromatography (HPLC–UV) analysis

We evaluated the HPLC analysis of AEPAS by a chromatographic system (N 2000, Korea) utilizing an Autosampler (YL 9150) with 100 μl fixed loop and a YL9120 UV–visible detector. We did the separation on an SGE Protocol PC18GP120 (250 mm × 4.6 mm, 5 μm) column at 25 °C. The mobile phase contailed methanol to water (70:30 v/v), and we utilized the isocratic mode; elution was achieved at a flow rate of 1 ml/min. The samples were run for 15 min and absorbance was read at 254 nm. The chromatographic data were assessed using the Autochro-2000 software.

### Total phenolic content determination

The technique described by Singleton et al.^54^ was used to determine the phenolic content. 200 µl of AEPAS at varying concentrations of 15–240 µg/ml was added to test tubes containing 2 ml of NaHCO_3_. 200 µl of Folin-Ciocalteu (Folin-C) reagent was added two minutes later, after which the mixtures were well mixed and incubated in the water bath for 30 min at 50 °C. We read the absorbance at 760 nm. The standard gallic acid was prepared in the same way as the AEPAS stock solution. The phenolic content was determined using the same protocol as used for AEPAS with the standard being used in place of AEPAS.

### Total flavonoid content determination

The aluminum chloride (AlCl_3_) procedure was used to determine the total flavonoid content of AEPAS^55^. The stock solutions of AEPAS were prepared at concentrations of 15–240 µg/ml and 1 ml of AEPAS was measured into clean tubes, after which 3 ml of distilled water and 0.3 ml of 5% NaNO_2_ were added. 0.3 ml of 10% AlCl_3_ and 2 ml of 1 M NaOH were added 5 min later and the volume of the solution in the test tube was diluted to 10 ml by adding distilled water. We read the absorbance at 510 nm. Quercetin was used as a reference and prepared by dissolving 4 mg in 1 ml of methanol. Various concentrations were prepared, and we assayed the flavonoid content using the same method outlined above for AEPAS.

### Assessment of in vitro antioxidant capacity

#### Total antioxidant activity (TAC)

The protocol described by Prieto et al.^56^ was used to determine the TAC. 1 ml of the reagent (28 mM sodium phosphate, 4 mM ammonium molybdate, and 0.6 M sulfuric acid) and 100 µl (0.1 ml) of AEPAS at various concentrations were placed in test tubes. The test tubes were then capped using foil paper and incubated in a water bath at 95 °C for about 90 min. The samples were then cooled, and we read the absorbance at 695 nm.

#### Assay of DPPH radical scavenging ability

We adopted the methodology described by Oboh and Rocha^57^ to determine the 2, 2- diphenyl-1-picrylhydrazyl scavenging effect of AEPAS. In brief, the extract (1 ml) at concentrations ranging from 15 to 240 µg/ml was added to 0.4 mM of DPPH solution prepared with methanol. Before reading, we kept the solution in the dark for 30 min, after which we read the absorbance of the mixture at 517 nm. This was done in triplicate. The reference used was ascorbic acid. The percentage of DPPH discoloration was then further calculated.

#### Assay of NO radical scavenging ability

The method described by Jagetia and Baliga^58^ estimated the capacity of nitric oxide to scavenge radicals in AEPAS. Varying concentrations (15–240 µg/ml) of AEPAS and the reference were prepared. 2.5 ml of 10 mM sodium nitroprusside (SNP) prepared in PBS (phosphate buffered saline) was added to 0.5 ml of varying concentrations of AEPAS and standard. The mixture was then further incubated for 150 min at 25 °C. A 0.5 ml aliquot was removed after the incubation period, and 0.5 ml of Griess reagent, which contains 1% (w/v) of sulphanilamide, 2% (v/v) of H_3_PO_4_, and 0.1% (w/v) of naphthylethylenediamine dihydrochloride, was added. We used 2 ml of sodium nitroprusside in PBS as the reference sample. The NO scavenging radical activities of AEPAS and gallic acid were both calculated and expressed as percentages.

#### Reducing ability

The reducing ability of AEPAS was assessed using Pulido et al.’s procedure^59^. We dissolved 2.5 ml of the sample (powdered) in about 2.5 ml of 200 mM sodium phosphate buffer of pH 6.6 and about 2.5 ml of 1% potassium ferricyanide. The mixtures were further incubated for 20 min at 50 °C, and then about 2.5 ml of 10% TCA was introduced to terminate the reaction. This was centrifuged for 10 min at 3000×*g* after which we added about 5 ml of the supernatant, an equivalent volume of water, and about 1 ml of 0.1% ferric chloride. The procedure followed above was also repeated, but in this case, it was for ascorbic acid, which was used as the reference sample and the reaction absorbance was read at 700 nm, followed by the calculation of the reducing ability which was expressed as an ascorbic acid equivalent.

### α-Amylase inhibitory activity

The inhibitory activity of *α*-amylase in AEPAS was determined following Shai et al.’s protocol^60^. 250 µl of the sample at various concentrations ranging from 15 to 240 µg/ml was then incubated along with about 500 µl of 2 U m/l porcine pancreatic amylase in 100 mmol/l of phosphate buffer with a pH of 6.8 for 20 min at 37 °C. Then, 250 µl of starch (about 1%) dissolved in phosphate buffer of pH 6.8 (100 mmol/l) was added to the mixture and kept warm for 1 h at 37 °C. 1 ml dinitrosalicylic acid containing (3,5-dinitrosalicylic acid (1%), phenol (about 0.2%), Na_2_SO_3_ (0.05%) and sodium hydroxide (1%)) color reagent was further added; after which we heated it for 10 min at 100 °C. A cold water bath was then used to cool down the mixture to about 25 °C, then the absorbance of the resulting mixture was read on the spectrophotometer at 540 nm. The standard used for this study was acarbose. The result was calculated and expressed as a percentage.

### α-Glucosidase inhibitory activity

The inhibitory activity of *α*-glucosidase by AEPAS was determined via a procedure provided by Nguelefack et al^61^. 1 mg of α-glucosidase was dissolved in a phosphate buffer (100 ml) at a pH of about 6.8 and containing 200 mg of bovine serum albumin (BSA). The mixture, which contained 10 µl of the sample at various ranges of concentration of about 15–240 µg/ml was remixed with 490 µl of phosphate buffer with a pH of about 6.8 and 250 µl of 5 mM p-nitrophenyl α-d-glucopyranoside. Pre-incubation for 15 min at 37 °C, after which there was an addition of 2000 µl Na_2_CO_3_ (200 mM) to stop the reaction. The α-glucosidase activity was estimated using a spectrophotometer to take the reading at 400 nm. The positive control used was acarbose of α-glucosidase inhibitor.

### Experimental animals

36 males experimental Wistar rats (245.54 ± 10.52 g) were bought from the Animal Holding Unit of the Department of Biochemistry, University of Ilorin, in Ilorin, Kwara State. The experimental animals were kept in cages (temperature: 25 ± 27 °C; as well as a photoperiod time of 12 h natural light and 12 h darkness; relative humidity between 40–45%. They were fed with animal feed (Top Feeds, Beside First Bank Plc, Adebayo, Ado-Ekiti, Nigeria) and freely available tap water. The animals were acclimatized for 14 days before we initiated the experiment.

### Dosage determination

Ethnobotanical survey and personal communications with traditional medicine practitioners revealed that around 200 ml of the juice extract is administered two times in a day for effective treatment of DM by a patient weighing about 70 kg. However, 18.46 g of AEPAS extract was derived after freeze-drying 200 ml of the juice. From this extrapolation, 53.3 mg/kg BWt was adopted as a dosage to determine the acclaimed anti-hyperglycemic potential of the extract.

### Diabetes mellitus induction

Induction of DM in rats was employed via the method described Ojo et al.^17^ Male Wistar rats (36) weighing 245.54 ± 10.52 g were fasted without food and given only water for 12 h. The fasting blood sugar level of the rats was checked and recorded before the administration of alloxan. Afterward, 30 male Wistar rats were given a 65 mg/kg single injection (I.P.) of alloxan dissolved in normal saline (NaCl) and a 5% glucose solution to induce insulin resistance. We checked the fasting blood sugar level for each DM-induced animal after 72 h, to authenticate the induction of diabetes mellitus. Animals having an FBS level > 250 mg/dl were diabetic.

### Animal groups and treatment with extract

Male Wistar rats (36) were selected and put into groups of six containing six rats each. The groupings were:

- Group 1: Normal rats + distilled water
- Group 2: Diabetic untreated rats
- Group 3: Diabetic rats + 30 mg/kg BWt of metformin (oral gavage)
- Group 4: Diabetic rats + 26.7 mg/kg BWt of AEPAS
- Group 5: Diabetic rats + 53.3 mg/kg BWt of AEPAS
- Group 6: Diabetic rats + 106.6 mg/kg BWt of AEPAS

The experiment lasted 14 days.

### Ethical approval

All experimental rats that were used for this study were all handled in line with the rules and regulations set aside for animal management used in research as contained in the manual prepared for the care and use of animals in a laboratory. In addition, the Landmark University ethical committee approved this research and gave an approval number LUAC/2021/005A in compliance with ARRIVE guidelines.

### Organ harvesting and analysis of samples

This research study lasted 14 days, after which we euthanized the rats using halothane. Then, the liver and pancreas were harvested and homogenized in a cold phosphate buffer, before being kept at a temperature of − 4°. The homogenized liver and pancreas were both centrifuged at 3000×*g* for 10 min to obtain a solution clear enough to evaluate some selected oxidative stress biomarkers. The blood was also collected and left for 1 h before centrifuging for 10 min at 3000×*g* to obtain a clear solution. We utilized this blood to determine the selected biochemical parameters.

### Biochemical indices

We determined serum insulin concentration based on the method described by Ojo et al.^17^, which uses an ELISA kit from Sweden in a multiple plate ELISA reader (Winooski, Vermont, in the USA). We evaluated serum total cholesterol using the protocol from Fredrickson et al.^62^ Triglyceride was determined using the procedure described by Tietz^63^. Jacobs et al.’s^64^ protocol was used to evaluate the HDL-cholesterol. Both LDL and VLDL-cholesterol were evaluated using Friedewald et al.’s procedure^65^. The atherogenic index (AI) was determined using Liu et al.’s method^66^. Then the coronary artery index (CRI) was estimated utilizing Wilson and Islam’s process^67^.

### Insulin resistance and β-cell function scores

Homeostasis model assessment of insulin resistance (HOMA-IR) was evaluated using Wilson and Islam’s method^67^. The homeostasis model assessment of the β-cell score (HOMA-β) was estimated using the equations utilized by Wilson and Islam^67^.

$$HOMA{-}IR = \frac{{\left[ {insulin\,\left( {\frac{{\text{U}}}{{\text{L}}}} \right) \times blood\,glucose\left( {\frac{{{\text{mmol}}}}{{\text{L}}}} \right)} \right]}}{{22.5}},$$

$$HOMA - \beta  = \frac{{\left[ {20 \times insulin\left( {\frac{{\text{U}}}{{\text{L}}}} \right)} \right]}}{{\left[ {blood\, glucose\,\left( {\frac{{{\text{mmol}}}}{{\text{L}}}} \right) - 3.5} \right]}}.$$

The conversion factors for units: insulin (1 U/l = 7.174 pmol/l) and blood glucose (1 mmol/l = 18 mg/dl).

### Determination of biomarkers of oxidative stress

The supernatants of both the liver and pancreas were used to assay for reduced glutathione (GSH) level^68^, GPx^69^, catalase (CAT)^37^, and superoxide dismutase (SOD) activities^70^, and MDA level^71^.

### Determination of the activities of glycolytic enzymes and glycogen level

The liver supernatant was used to analyze the activities of the glycolytic enzymes, including hexokinase^72^, glucose-6-phosphatase (G6Pase)^73^, and fructose 1,6-bisphosphatase^74^. We estimated the liver glycogen following the procedure described by Morales et al.^75^.

### Determination of inflammatory biomarkers

TNF-α, IL-6, and NF-κB, were determined in the serum utilizing the procedure delineated in ELISA (Sigma Chemical Company Inc. (St. Louis, MO, USA)) kits.

### Total RNA isolation

We removed total RNA from entire organs following a technique described by Omotuyi et al.^76^ The organs were homogenized in cool (4 °C) TRIzol reagent (Zymo Research, USA, Cat:R2050-1-50, Lot: ZRC186885). Absolute RNA was apportioned in chloroform solvent (BDH Analytical Chemicals, Poole, England Cat: 10076-6B) and centrifuged at 15,000 rpm for 15 min (Abbott Laboratories, Model: 3531, Lake Bluff, Illinois, United States). The RNA from the supernatant was precipitated using an equivalent amount of isopropanol (Burgoyne Urbidges & Co, India, Cat: 67-63-0). The RNA pellet was washed two times in 70% ethanol (70 ml absolute ethanol (BDH Analytical Chemicals, Poole, England Cat: 10107-7Y) in 30 ml of nuclease-free water (Inqaba Biotec, West Africa, Lot no: 0596C320, code: E476-500ML)). The pellets were air-dried for 5 min and solubilized in RNA buffer (1 mM sodium citrate, pH 6.4).

### cDNA transformation

Before cDNA transformation, absolute RNA amount (concentration (µg/ml) = 40 * A_*260*_) and quality (≥ 1.8) were evaluated using the proportion of A_260_/A_280_ (A = absorbance) read using a spectrophotometer (Jen-way UV–VIS spectrophotometer model 6305, UK). DNA impurity detached from RNA was separated after DNAse I treatment (NEB, Cat: M0303S) as described by the manufacturer. A 2 µl solution containing 100 ng DNA-free RNA was changed to cDNA by employing the M-MuLV Reverse Transcriptase Kit (NEB, Cat: M0253S) in 20 µl final volume (2 µl, N^9^ random primer mix; 2 µl, 10X M-MuLV buffer; 1 µl, M-MuLV RT (200 U/µl); 2 µl, 10 mM dNTP; 0.2 µl, RNase Inhibitor (40 U/µl) and 10.8 µl nuclease-free water). The reaction continued at 25 °C O/N. Inactivation of M-MuLV Reverse transcriptase was achieved at 65 °C after 20 min.

### PCR amplification and agarose gel electrophoresis

PCR intensification for the assessment of genes whose primers (Primer3 software) are recorded below were performed using the accompanying procedure: PCR enhancement was achieved in a 25 µl volume mixture containing 2 µl cDNA (10 ng), 2 µl primer (100 pmol) 12.5 µl Ready Mix Taq PCR master mix (One Taq Quick-Load 2x, master mix, NEB, Cat: M0486S) and 8.5 µl nuclease-free water. Early denaturation at 95 °C for 5 min was followed by 20 cycles of amplification (denaturation at 95 °C for 30 s, annealing for 30 s and amplified at 72 °C for 60 s), concluding with a final amplification at 72 °C for 10 min. In all the tests, we incorporated negative controls, in which the mixture had no cDNA. The amplicons were separated on 1.5% agarose gel (Cleaver Scientific Limited: Lot: 14170811) in Tris (RGT reagent, China, Lot: 20170605)-Borate (JHD chemicals, China, Lot 20141117)-EDTA buffer (pH 8.4).

PI3K mRNA Sequence (5′→3′)

- Forward primerGGTGCTAAGGAGGAGCACTG
- Reverse primerCCATGTGGTACAGGCCAGAG

AKT mRNA Sequence (5′→3′)

- Forward primerAAGGACCCTACACAGAGGCT
- Reverse primerAAGGTGGGCTCAGCTTCTTC

GAPDH mRNA Sequence (5′→3′)

- Forward primerGCATCTTCTTGTGCAGTGCC
- Reverse primerGAGAAGGCAGCCCTGGTAAC

Bcl-2 mRNA Sequence (5′→3′)

- Forward primerGCGTCAACAGGGAGATGTCA
- Reverse primerTTCCACAAAGGCATCCCAGC

PCNA mRNA Sequence (5′→3′)

- Forward primerAGCAACTTGGAATCCCAGAACA
- Reverse primerCACAGGAGATCACCACAGCA

Cyclophilin A mRNA Sequence (5′→3′)

- Forward primerTGGAGAGCACCAAGACAGACA
- Reverse primerTGCCGGAGTCGACAATGAT

### Amplicon image processing

In-gel amplicon bands pictures were caught on camera analyzed using the Keynote platform according to Omotuyi et al.’s procedure^76^, and evaluated using image-J software.

#### Histology

After paraffin embedding, the fixed pancreatic tissues were stained with hematoxylin and eosin. A Leica slide scanner was used to view the slides (SCN 4000, Leica Biosystems, Germany).

### Data analysis

We conducted the in vitro studies in triplicate. In the in vivo analysis, the data were all interpreted as mean ± SEM for six readings across the groups. We then subjected the data from this study to analysis by using one-way analyses of variance (ANOVA). Tukey’s post hoc comparison test was done using GraphPad Prism 9 version, and the significance level was set at *P* < 0.05.

## Supplementary Information

Supplementary Figures.

## Data Availability

Data are available on request.
