# Supplementary material for: Antidiabetic activity of avocado seeds (Persea americana Mill.) in diabetic rats via activation of PI3K/AKT signaling pathway
Source: Sci Rep. 2022 Feb 21;12:2919. doi: 10.1038/s41598-022-07015-8 (PMC8861005; doi:10.1038/s41598-022-07015-8)
Supplement: Supplementary file 1 — Supplementary Figures. [file 41598_2022_7015_MOESM1_ESM.docx]

**Antidiabetic activity of Avocado seeds (*Persea americana* Mill.) in diabetic rats via activation of PI3K/AKT signaling pathway**

**Oluwafemi Adeleke Ojo^1*^, Jennifer Chidubem Amanze^1^, Abosede itunuoluwa Oni^1^, Susan Grant^1^, Matthew Iyobhebhe^1^, Tobiloba Christiana Elebiyo^1^, Damilare Rotimi^1^, Nnaemeka Tobechukwu Asogwa^2^, Babatunji Emmanuel Oyinloye^3^, Basiru Olaitan Ajiboye^4^, Adebola Busola Ojo^5^**

^1^ Phytomedicine, Molecular Toxicology and Computational Biology Research Group,

Department of Biochemistry, Landmark University, Omu-Aran, Nigeria

^2^ Central Research Laboratory 123B, University Road, Tanke Ilorin, Nigeria

^3^ Department of Biochemistry, Afe Babalola University Ado-Ekiti, Nigeria

^4^ Department of Biochemistry, Federal University Oye-Ekiti, Nigeria

^5^ Department of Biochemistry, Ekiti State University, Ado-Ekiti, Nigeria


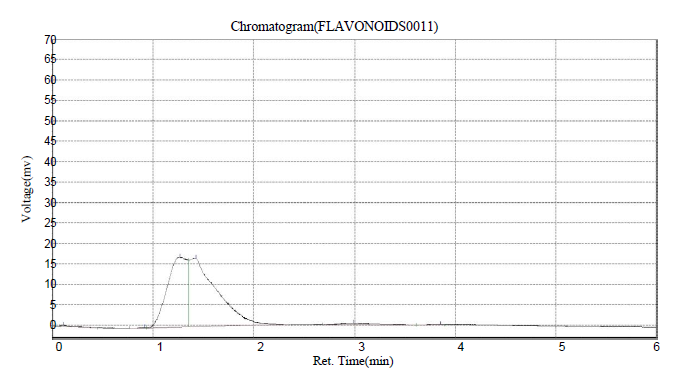


**Figure S1**: HPLC chromatogram of AEPAS at 254 nm

Legend: AEPAS: aqueous extract of *Persea americana* seeds


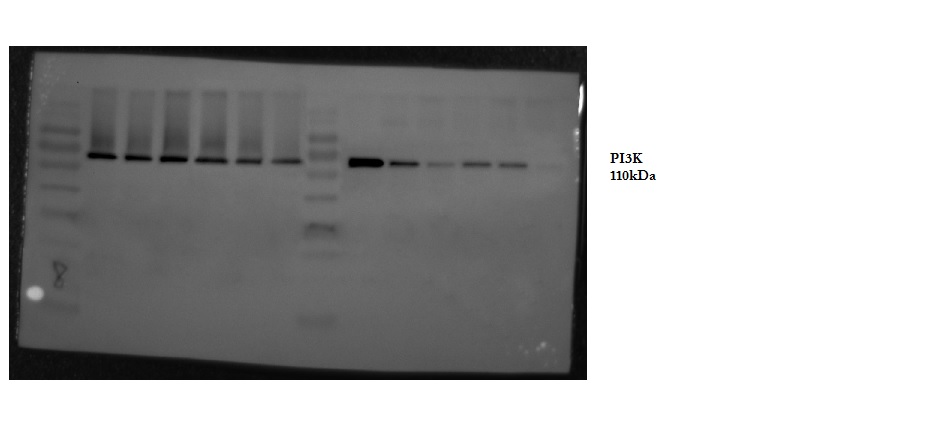


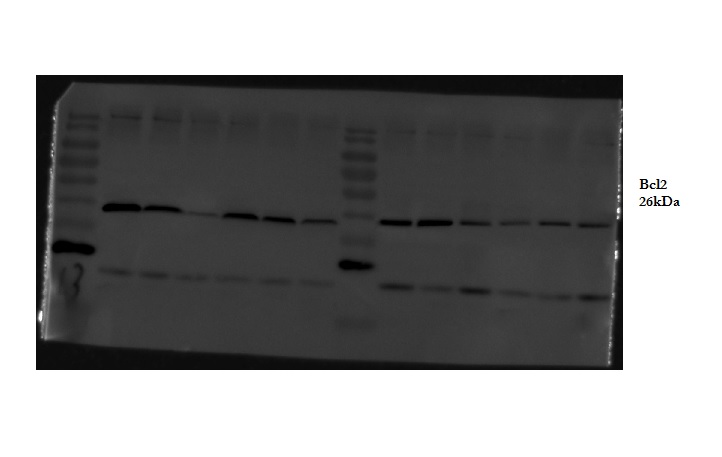

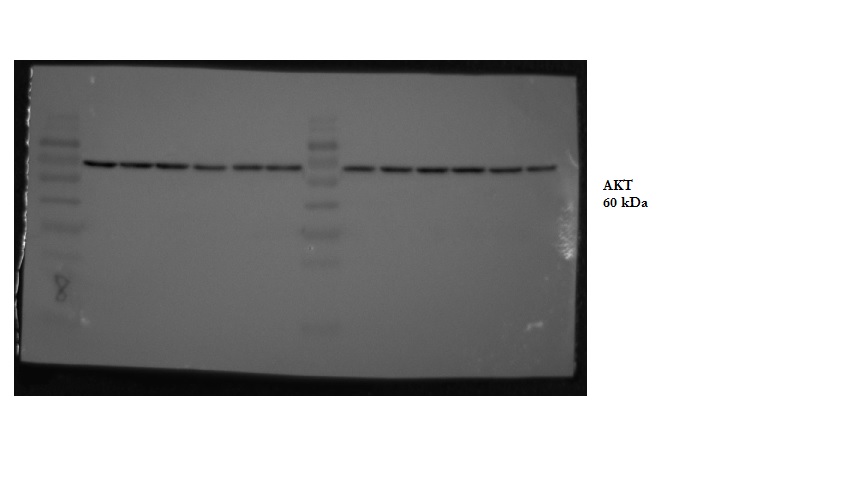


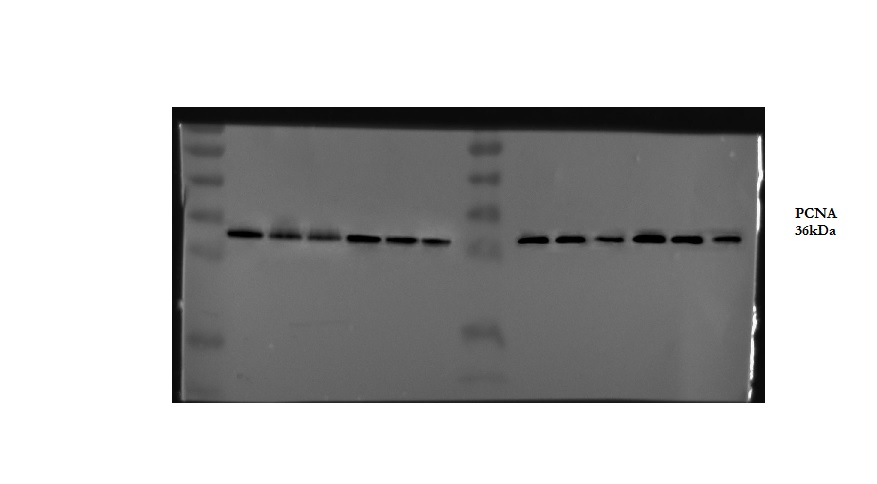


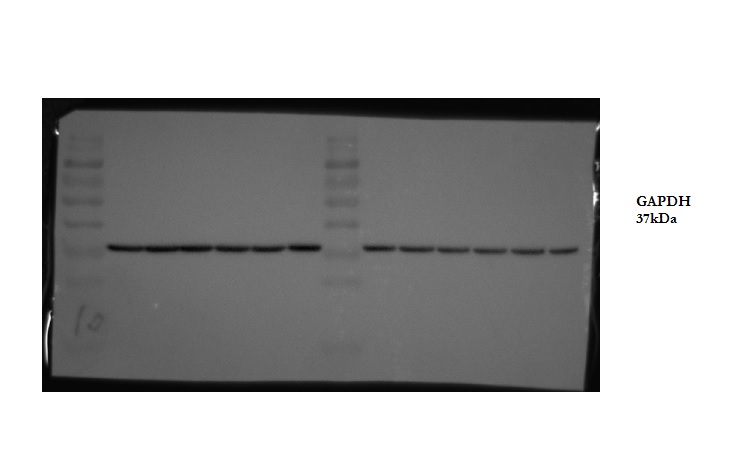


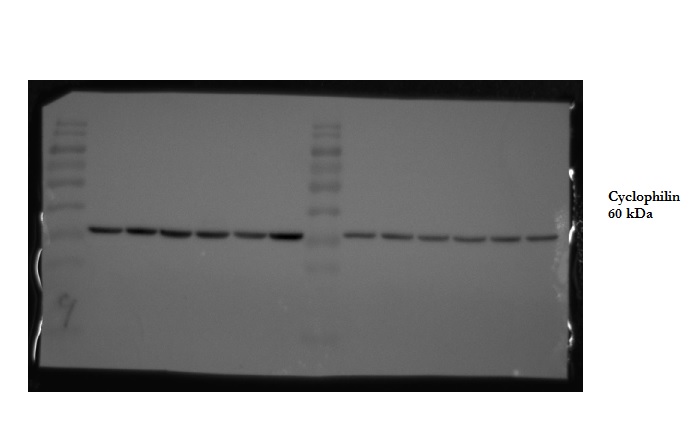


**S2**: Supplementary figure for the original source of Western blot images
